# The Story of the Insane from Year to Year

**Published:** 1897-02-20

**Authors:** 


### THE STORY OF THE INSANE FROM YEAR TO YEAR.

Ninth Series.

### DORSET COUNTY ASYLUM.

The numbers rose from 506 to 610. There were 179 admissions, 29 readmissions, 37 recoveries, and 50 deaths. The percentage of recoveries stands at 31·9, and that of the deaths at 8·78. Intemperance in drink is the cause of insanity in 14 of the admissions, and hereditary tendency in 37. Of the deaths 19 were caused by cerebral diseases and 8 by consumption. Dr. MacDonald finds that hereditary tendency to insanity exists in upwards of 30 per cent. of the admissions, and he adds "that the marriage of individuals predisposed to mental disease must lead to a deterioration of the race by the production of ill-developed, malformed, and defective children." There is no doubt, whatever about this, and instances of marriage following release from asylums are so common that they have ceased to be remarkable. Dr. MacDonald seems to think it would be impossible to restrict the liberty of the subject in such matters; but surely it is licence rather than liberty, and in any case it would be better to curtail liberty a little rather than condemn innocent children to a life of misery. The old asylum at Forston has been abandoned and a new one has been built for female patients. This is being worked on the open-door system, and we are pleased to learn that this has been extended to the male asylum. The visitors say that "Dr. MacDonald has continued to give unwearied attention to the many requirements of the asylum." A ground plan of the new building is given. Private patients pay from 10s. to two guineas a week. The charge for in-county paupers is 8s. 6d. per head per week.

